# Endoplasmic Reticulum Stress Signaling Is Involved in Mitomycin C(MMC)-Induced Apoptosis in Human Fibroblasts via PERK Pathway

**DOI:** 10.1371/journal.pone.0059330

**Published:** 2013-03-22

**Authors:** Kun Shi, Daode Wang, Xiaojian Cao, Yingbin Ge

**Affiliations:** 1 Department of Orthopedics, The First Affiliated Hospital of Nanjing Medical University, Nanjing, People’s Republic of China; 2 Department of Physiology, Nanjing Medical University, Nanjing, People’s Republic of China; Osaka University Graduate School of Medicine, Japan

## Abstract

Endoplasmic reticulum (ER) stress-mediated cell apoptosis has been implicated in various cell types, including fibroblasts. Previous studies have shown that mitomycin C (MMC)-induced apoptosis occurs in fibroblasts, but the effects of MMC on ER stress-mediated apoptosis in fibroblasts have not been examined. Here, MMC-induced apoptosis in human primary fibroblasts was investigated by exposing cells to a single dose of MMC for 5 minutes. Significant inhibition of cell proliferation and increased apoptosis were observed using a cell viability assay, Annexin V/propidium iodide double staining, cell cycle analysis, and TUNEL (terminal deoxynucleotidyl transferase dUTP nick-end labeling) staining. Upregulation of proapoptotic factors, including cleaved caspase-3 and poly ADP-ribose polymerase (PARP), was detected by Western blotting. MMC-induced apoptosis was correlated with elevation of 78-kDa glucose-regulated protein (GRP78) and C/EBP homologous protein (CHOP), which are hallmarks of ER stress. Three unfolded protein response (UPR) sensors (inositol-requiring enzyme 1, IRE1; activating transcription factor 6, ATF6; and PKR-like ER kinase, PERK) and their downstream signaling pathways were also activated. Knockdown of CHOP attenuated MMC-induced apoptosis by increasing the ratio of BCL-2/BAX and decreasing BIM expression, suggesting that ER stress is involved in MMC-induced fibroblast apoptosis. Interestingly, knockdown of PERK significantly decreased ER stress-mediated apoptosis by reducing the expression of CHOP, BIM and cleaved caspase-3. Reactive oxygen species (ROS) scavenging also decreased the expression of GRP78, phospho-PERK, CHOP, and BIM. These results demonstrate that MMC-induced apoptosis is triggered by ROS generation and PERK activation.

## Introduction

The control of excessive fibrosis after laminectomy is one of the main concerns associated with spine surgery. The constrained roots are stretched, and their normal motion in the vertebral column is impeded by excessive fibrosis caused by scar formation, leading to intractable back and leg pain, which constitute the main features of ‘failed back surgery syndrome’ (FBSS) [1], [2], [3]. Furthermore, epidural adhesions make reexposure of the same operative area technically difficult and dangerous [4], [5].

Mitomycin C (MMC), is a potent reactive oxygen species (ROS)-generating antitumor drug used to treat cancer. In addition to being used in the intravenous treatment of esophageal carcinoma, gastric cancer, anal cancers, and breast cancers, MMC has been applied topically as an anti-fibrotic drug to inhibit fibroblast proliferation after glaucoma filtering surgery [6], [7] and to treat esophageal and tracheobronchial stricture [8], [9]. MMC has also been reported to prevent scarring during lumbar laminectomy and tendon surgery in a rat model [10], [11], [12]. Application of MMC may result in prevention of postoperative scarring by decreasing the production of fibroblasts and scar tissue.

MMC exhibits antitumor activity via ROS-dependent activation of apoptotic cell death, which involves mitochondrial dysfunction and caspase activation in tumor cells [13], [14], [15], [16]. MMC also induces cytotoxicity and modulates key regulators of apoptosis (caspases and c-Jun N-terminal kinase, JNK) in fibroblasts [17]. These effects are linked to the increased production of ROS in tumor cells. However, MMC treatment of fibroblasts differs from that of tumor cells, and additional studies are needed to determine the mechanism of MMC-induced apoptosis in fibroblasts.

The endoplasmic reticulum (ER) is a multifunctional organelle that is responsible for protein synthesis, folding and export, lipid biosynthesis, vesicular traffic and cellular calcium storage. The ER provides a unique oxidizing environment for the folding of and disulfide bond formation in proteins before their transit to the Golgi compartment. The unfolded protein response (UPR) is an adaptive signaling pathway designed to prevent the accumulation of misfolded proteins in the ER lumen and minimize the stress related to oxidative protein folding [18]. Chronic unresolved accumulation of unfolded proteins in the ER leads to apoptosis, which is mediated through the C/EBP homologous protein (CHOP) pathway [19], [20]. Cells showing compromised ER function, such as cells that are defective in the UPR or ER-associated protein degradation, are susceptible to ROS production [19], [21]. ER stress and oxidative stress are closely linked events, and ROS are an essential component of the events leading to protein misfolding in the ER and ER stress-induced apoptosis [22].

In a previous study, we demonstrated that topical MMC application could prevent epidural scar adhesions in adult rats after lumbar laminectomy in vivo [11], [12]. Another group showed that MMC induced the activation of apoptotic molecules, including JNK and caspase-3, in human Tenon’s capsule fibroblast cells [17]. In light of the excessive ROS production observed in MMC-treated cells, the aim of the present study was to explore the mechanism of ER stress-induced apoptotic cell death in MMC-treated fibroblasts. We examined the effect of 5 minutes of MMC exposure on the following activities: (1) apoptosis in primary fibroblasts, (2) the activity and function of UPR sensor proteins, and (3) the involvement of ROS in the ER stress-induced apoptosis pathway. Our findings showed that MMC treatment triggers ER stress, and UPR and PKR-like ER kinase (PERK) activation can initiate fibroblast apoptosis.

## Materials and Methods

### Ethics Statement

A primary fibroblast cell line was established from epidural scar fibroblasts isolated from patients who took part in a clinical study approved by the Ethical Committee of the First Affiliated Hospital of Nanjing Medical University in accordance with the provisions of the Declaration of Helsinki. The clinical trial registration number is ChiCTR-TRC-10001079. In this study, all patients who had suffered laminectomy surgery had to accept re-operation for new symptoms. Excess epidural scar tissue removed during surgery was used for cell culture. Written informed consent was obtained from all patients.

### Cell Culture

Fibroblasts were cultured at 37°C under 5% CO_2_ in Dulbecco’s modified Eagle’s medium (DMEM; Gibco, USA) with L-glutamine (2 mM), 10% fetal bovine serum (FBS; Gibco, USA), penicillin (100 IU/ml), and streptomycin (100 µg/ml) (Thermo, USA). Cells in exponential growth phase between passages 4 and 7 were used for all experiments.

### MMC Treatment

A fibroblast monolayer seeded in 24-well plates or 10 cm dishes overnight was washed with phosphate-buffered saline (PBS; pH 7.4) and pretreated with or without antioxidants including N-acetyl-L-cysteine (NAC), glutathione (GSH), and edaravone (Beyotime, Hangzhou, China) or a JNK inhibitor SP600125 (St. Louis, MO, USA). for 2 hours. The cells were then subjected to a single application of 0.4 mg/ml MMC (diluted in PBS; Kyowa Hakko Kogyo Ltd., Tokyo, Japan) for 5 minutes. The control groups were treated with PBS alone for 5 minutes. After treatment, the cells were immediately washed three times with PBS and maintained in growth medium for subsequent experiments.

### Cell Viability Assay

Cell viability was determined using the Cell Counting Kit-8 (CCK-8) (Dojindo Laboratories, Kumamoto, Japan) according to the manufacturer’s instructions. Fibroblasts were plated in 6 replicates in 96-well plates (100 µl, 2×10^3^/well). After plating (24 hours), the cells were subjected to various treatments. The CCK-8 solution (10 µl) was added to each well, and the cells were incubated for another 3 hours at 37°C, after which the optical density was measured at 450 nm using a microplate absorbance reader (Bio-Tek, Elx800, USA). Cells that stained positively with the CCK-8 solution were considered viable and are presented as a percentage compared with control cells.

### Annexin V/Propidium Iodide Double Staining and Cell Cycle Analysis

Annexin V/propidium iodide double staining and cell cycle analysis were used to detect apoptosis and the stages of the cell cycle, respectively. Fibroblasts were plated in 60-mm dishes (3 ml, 1×10^6^/well) and incubated for 24 hours at 37°C. After treatment with MMC, the detached and adherent cells were collected at different time points and washed twice with ice-cold PBS. The cells were then resuspended in binding buffer at a concentration of 1×10^6^ cells/ml and incubated with Annexin V-FITC and propidium iodide (BD Biosciences, USA) to achieved double staining, according to the manufacturer’s instructions. The mixture was incubated in the dark for 15 minutes at room temperature prior to analysis. For cell cycle analysis, the collected cells were fixed at −20°C in ice-cold 70% ethanol overnight. After two washes with PBS, the cells were stained with propidium iodide and analyzed using the Beckman Coulter FC500 flow cytometry system and CXP software (Beckman Coulter, Fullerton, CA).

### TUNEL Staining

TdT-mediated dUTP-biotin nick end labeling (TUNEL) (Roche, USA) was conducted to identify apoptotic fibroblasts. Cells were seeded in 6-well plates (2 ml, 5×10^5^ cells/well) and incubated overnight at 37°C to allow the cells to adhere. After treatment with 0.4 mg/ml MMC as described above, the cells were fixed in 4% paraformaldehyde for 30 minutes at room temperature. After rinsing with PBS, the cells were permeabilized with 0.1% Triton X-100 in 0.1% sodium citrate for 2 minutes on ice and incubated with the TUNEL reagent for 1 hour at 37°C in the dark. The cells were then rinsed twice with PBS and stained with 1 µg/ml DAPI (Beyotime, Hangzhou, China), which results in characteristic blue nuclear staining. Following staining, the apoptotic features of cell death were examined via fluorescence microscopy (Olympus BX 51, Tokyo, Japan). The obtained images were merged and analyzed using Image J software. The percentage of TUNEL-positive cells was defined as the number of TUNEL-stained cells divided by the number of DAPI-stained cells. At least 500 cells in 12 randomly selected fields of view from each well were counted by a naive observer.

### ROS Measurements

Changes in ROS levels were detected with 2′,7′-dichlorofluorescin diacetate (DCFH-DA) (KeyGEN Biotech, China). Cells were plated at a density of 1×10^6^ per 60 mm dish, exposed to 0.4 mg/ml MMC as described above, and then incubated for 24 hours. The cells were stained with 10 µM DCFH-DA for 20 minutes at 37°C in the dark. After two washes with PBS, the cells were analyzed via fluorescence microscopy (Olympus BX 51, Tokyo, Japan) or flow cytometry (Beckman Coulter, Fullerton, CA). The images were merged using Image J software. Cells showing green fluorescence were considered to be ROS positive. The mean fluorescence density indicating ROS generation was measured using flow cytometry.

### Lipid Peroxidation Assay

To quantify the lipid peroxidation level, malondialdehyde (MDA) synthesis was measured in a lipid peroxidation MDA assay (Beyotime, Hangzhou, China) according to the manufacturer’s instructions. Briefly, cells were plated in 10 cm dishes and subjected to various treatments for 24 hours after plating. The cells were harvested via trypsinization, and cellular extracts were prepared via sonication in ice-cold buffer (50 mM Tris–HCl, pH 7.5, 5 mM EDTA, and 1 mM DTT). The lysates were then centrifuged at 10,000×*g* for 15 minutes, and the supernatants were subjected to measurement of MDA levels and protein contents. For the MDA assay, a thiobarbituric acid (TBA) solution was added to supernatant samples, which were then placed in a boiling water bath for 15 minutes. After cooling to room temperature, sample absorbance was measured at 532 nm. The protein concentration was determined using the BCA Protein Assay Kit (Thermo, USA). The MDA levels are expressed as nanomoles of MDA per milligram of protein (nmol/mg protein).

### Western Blot Analysis

Fibroblasts were lysed on ice in lysis buffer (Beyotime, Hangzhou, China) according to the manufacturer’s instructions, and the lysates were centrifuged at 14,000×*g* at 4°C for 15 minutes. The protein concentration was determined using the BCA Protein Assay Kit (Thermo, USA). Equal amounts (25 µg/lane) of total protein were subjected to electrophoresis in a 10% SDS-polyacrylamide gel. Following electrophoresis, the proteins were electro-transferred to polyvinylidene difluoride membranes (Millipore, USA). The membranes were then blocked with 5% skim milk in TBST at room temperature for 2 hours and subsequently incubated with primary antibodies (diluted 1∶500 to 1∶1,000) at 4°C overnight. Anti-78-kDa glucose-regulated protein (GRP78), anti-CHOP, anti-cleaved caspase-3, anti-PERK, anti-phospho-PERK, anti-eukaryotic translation initiation factor 2α (eIF2α), anti-phospho-eIF2α (phospho S51), anti-poly ADP-ribose polymerase (PARP), anti-BIM, anti-BAX, and anti-BCL-2 antibodies were obtained from Cell Signaling Technology (Cell Signaling Technology, USA). An anti-β-actin antibody was obtained from Santa Cruz Biotechnology (Santa Cruz Biotechnology, USA). Anti-activating transcription factor 6 (ATF6), anti-inositol-requiring enzyme 1α (IRE1α), and anti-phospho-IRE1α (S724) antibodies were purchased from Abcam (Abcam, USA). The membranes were next washed three times in TBST and incubated with horseradish peroxidase-conjugated goat anti-mouse or anti-rabbit IgG (Santa Cruz Biotechnology, USA) (diluted 1∶5,000) for 1 hour. The immune complexes were visualized via fluorography using an enhanced ECL system (Millipore, USA).

### Reverse Transcriptase PCR and Quantitative Real-time PCR Analysis

Cells were harvested at various time points after treatment with MMC (0.4 mg/ml). For all treatments, total RNA was extracted with TRIzol reagent (Invitrogen, USA) according to the manufacturer’s instructions. First-strand cDNA was synthesized using random primers (Invitrogen, USA). The primers in the reverse transcriptase (RT)-PCR analysis were as follows: human X-box binding protein 1 (XBP-1) mRNA, 5′-TTACGAGAGAAAACTCATGGC-3′ (sense) and 5′-GGGTCCAAGTTGTCCAGAATGC-3′ (anti-sense); human GAPDH mRNA, 5′-TGAACGGGAAGCTCACTGG-3′ (sense) and 5′-TCCACCACCCTGTTGCTGTA-3′ (anti-sense). The reaction conditions consisted of the following steps: 95°C for 5 minutes, 95°C for 1 minute, 58°C for 30 seconds, 72°C for 30 seconds, and 72°C for 5 minutes, with 35 amplification cycles. The obtained PCR products were resolved in a 2% agarose/1×TAE gel, followed by visualization under UV light. Real-time quantitative RT-PCR was performed using the ABI Prism 7000 sequence detection system (Applied Biosystems) and SYBR Green PCR Master Mix (Applied Biosystems, USA). The following primers were employed in these reactions: human CHOP mRNA, 5′-ACCAAGGGAGAACCAGGAAACG-3′ (sense) and 5′-TCACCATTCGGTCAATCAGAGC-3′ (anti-sense); human GRP78 mRNA, 5′-mRNA, CGGGCAAAGATGTCAGGAAAG-3′ (sense) and 5′-TTCTGGACGGGCTTCATAGTAGAC-3′ (anti-sense); and human GAPDH mRNA, 5′-GGGCTCTCCAGAACATCATCC-3′ (sense) and 5′-GTCCACCACTGACACGTTGG-3′ (anti-sense). The relative mRNA levels for all genes were normalized to the levels of GAPDH.

### Gene Silencing Using Small Interfering RNAs (siRNAs) or via Lentiviral Infection

Fibroblasts were plated in 60-mm wells and allowed to reach 50% confluence at the time of transfection. CHOP and non-targeting control siRNAs were purchased from Santa Cruz Biotechnology. Cells were transfected with 100 nM concentrations of siRNAs diluted in Opti-Eagle’s minimal essential medium (MEM) using Lipofectamine 2000 (Invitrogen, USA), according to the manufacturer’s instructions. The cells were treated with MMC at 48 hours post-transfection for subsequent experiments.

Lentiviral vectors that contained target genes including PERK, IRE1, and ATF6 were purchased from Shanghai Genechem Co. Ltd. (Genechem, China). Lentiviral infection was carried out according to the manufacturer’s instructions. Fibroblasts were incubated in growth media with the lentiviruses at an MOI of 20 in the presence of 2 µg/ml polybrene (Gibco, USA) overnight, after which the medium was removed, and fresh complete medium was added. Following transfection (48 hours), the cells were grown in culture with 2 µg/ml puromycin (Sigma, USA) for at least 96 hours to select for stably transfected cells for subsequent experiments.

### Statistical Analysis

Data are presented as the mean ±SD of triplicate experiments. Significant differences between treatment groups were analyzed via one-way ANOVA, followed by a Dunnett test or Student’s *t*-test using SPSS (Statistical Package for the Social Sciences) 13.0 software. Statistical significance was defined as a P value <0.05.

## Results

### Apoptotic Effect of MMC

**Figure 1:**
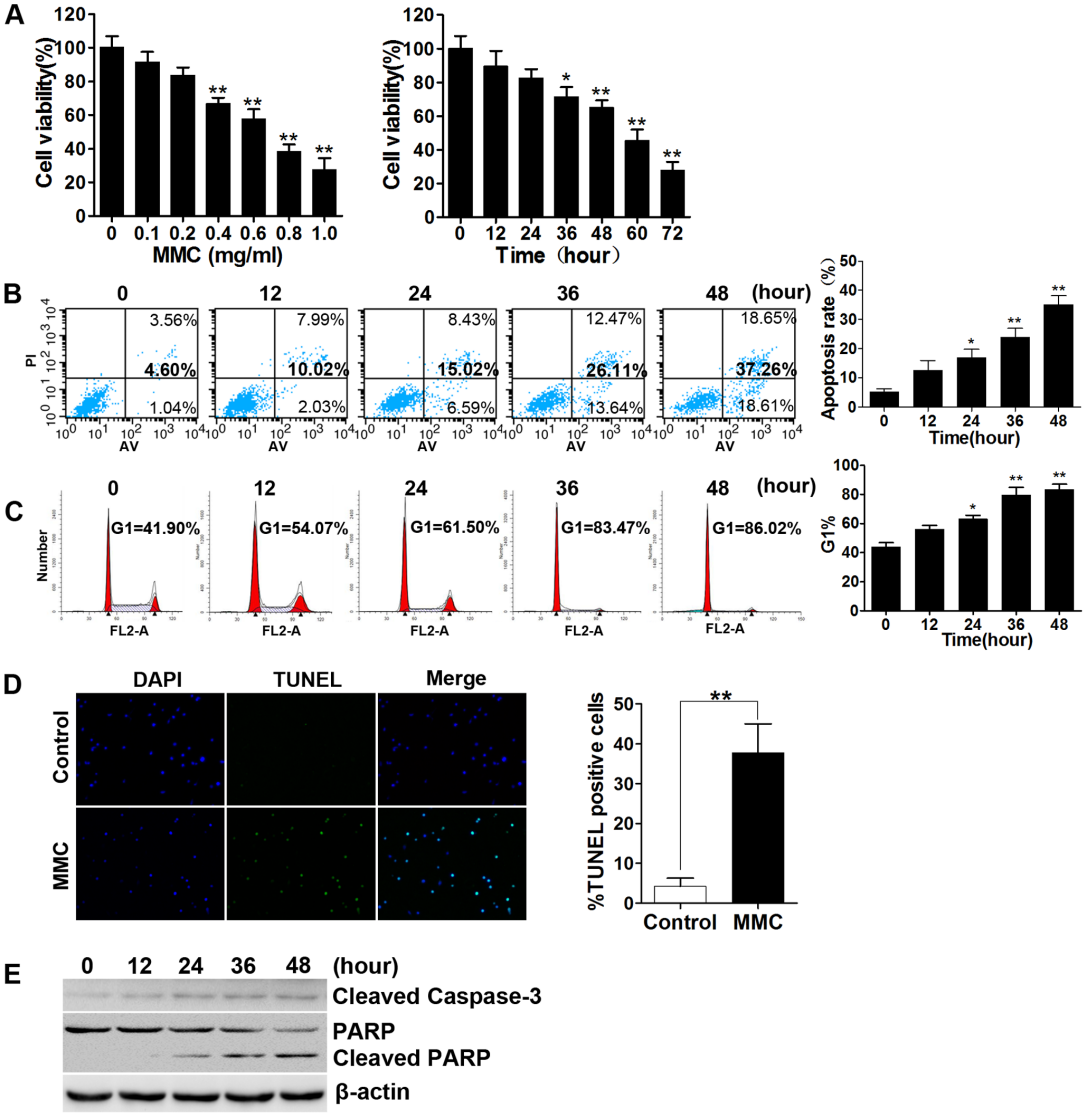
Application of MMC for 5 minutes induces apoptosis in human fibroblasts. (A) Cell viability after different treatments was detected by using the CCK-8 assay. MMC-induced growth inhibition occurred in a dose- and time-dependent manner. (B) Apoptosis analysis was assessed via Annexin V/propidium iodide double staining. Cells were exposed to 0.4 mg/ml MMC for 5 minutes and then incubated for the corresponding indicated times. Apoptosis rates were determined via flow cytometry analysis. The cells shown in the bottom right quadrant were Annexin V-FITC positive and propidium iodide negative, indicating an early stage of apoptosis. The cells in the top right quadrant stained positively for Annexin V-FITC and propidium iodide, indicating that they consisted of secondary late apoptotic/necrotic cells. The total percentage of cell death is shown in bold. Statistical analysis of the total recorded apoptotic cells was performed, and the results are shown in the bar graphs. (C) Cell cycle analysis was conducted to detect the changes in the cell cycle at different time points after MMC treatment (0.4 mg/ml, 5 minutes). (D) Cells were TUNEL stained 48 hours after MMC treatment and then observed using a fluorescence microscope. Nuclei are shown in blue, and TUNEL staining is shown in green. (E) Western blots revealed that MMC induces cleavage of caspase-3 from a size of 35 kDa to 17 kDa and the degradation of poly ADP-ribose polymerase (PARP) by the cleaved caspase-3 into 85 to 90 kDa fragments. β-actin was included as a control. Gels were run in triplicate. The histograms in panels A, B, C and D represent the mean ±SD of three independent experiments. *P<0.05, **P<0.01 versus 0 or the control group.

We initially examined the effect of MMC on fibroblasts. Cells were exposed to various concentrations (0–1.0 mg/ml) of MMC for 5 minutes, after which the medium was removed, and the cells were maintained in fresh medium for 48 hours. Additionally, cells were treated with 0.4 mg/ml MMC for 5 minutes were incubated for different time periods. To confirm whether MMC affects fibroblast viability, CCK-8 assays were performed. MMC induced a decrease in fibroblast viability in a dose- and time-dependent manner. The ED50 was estimated to be 0.4 mg/ml. A marked reduction in cell viability was observed 36 hours after treatment with MMC at a concentration of 0.4 mg/ml (Fig. 1A). Similar results were observed using Annexin V/propidium iodide double staining. Treatment of cells with 0.4 mg/ml MMC for 12, 24, 36, or 48 hours caused approximately 2.03%, 6.59%, 13.64%, and 18.61% early stage cell apoptosis, respectively (bottom right quadrant). The total percentage of apoptosis (bottom and top right quadrant) also increased gradually over time (Fig. 1B). Cell cycle analysis revealed that the cells were blocked at the G1/S transition after a 24-hour incubation (Fig. 1C). TUNEL staining was used to detect DNA fragmentation, which is one of the hallmarks of apoptosis. As expected, the numbers of TUNEL-positive cells (stained with green fluorescence) markedly increased 48 hours after MMC treatment (Fig. 1D). Next, we detected the activated form of caspase-3 and its intracellular substrate PARP, both of which are considered biochemical markers of apoptosis [23]. Significantly increased cleavage of caspase-3 and degradation of PARP by caspase-3 into 85 to 90 kDa fragments were observed after 24 hours of incubation (Fig. 1E). These results suggest that treatment with MMC reduces fibroblast viability via apoptosis.

### ER Stress Induced by MMC

**Figure 2:**
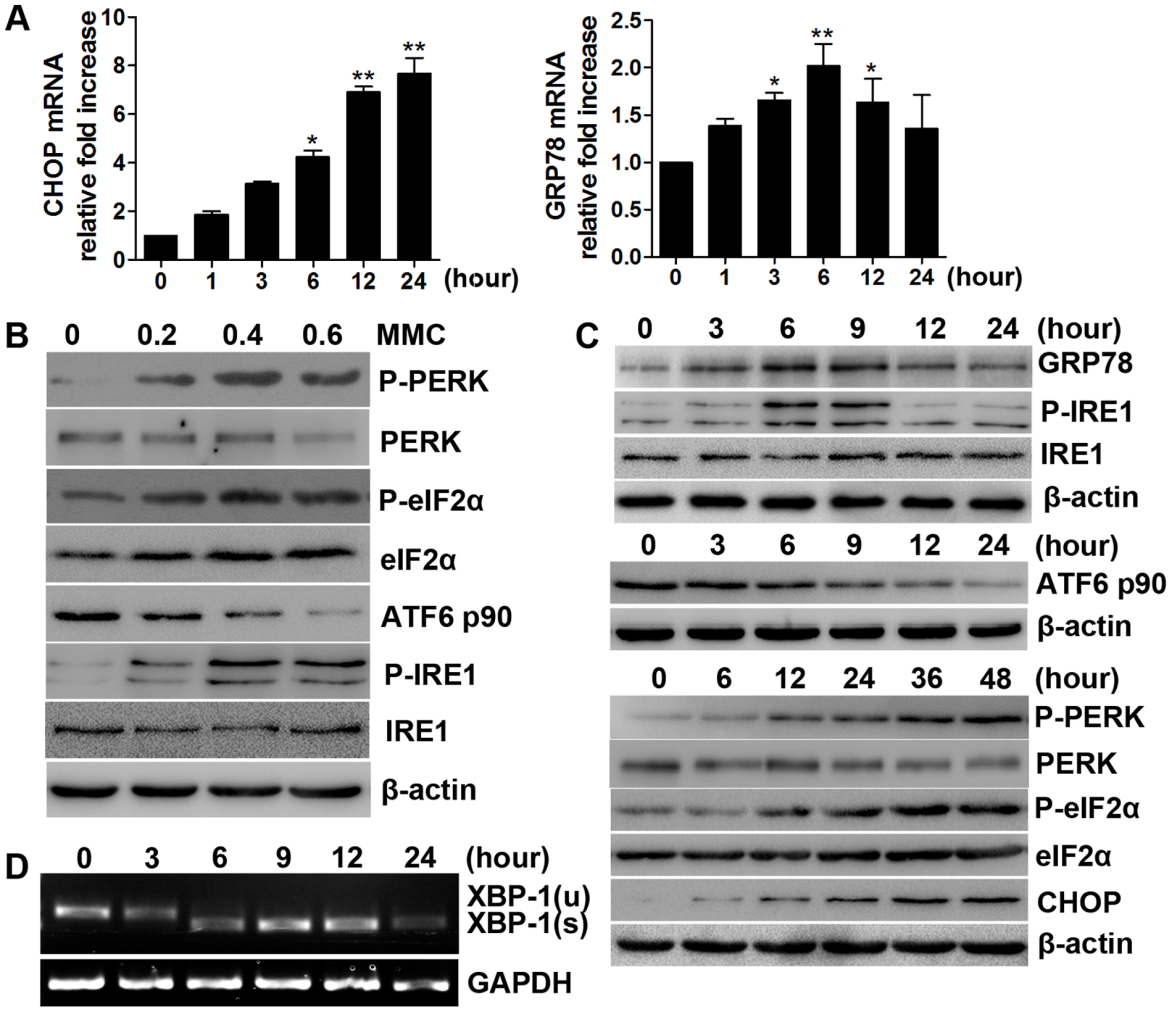
MMC induces ER stress and the UPR. (A) Relative CHOP and GRP78 mRNA levels were detected by quantitative real-time PCR and normalized to GAPDH mRNA levels. These values were compared with the 0 hour group. The values represent the mean ± SD of three separate experiments. *P<0.05, **P<0.01. (B-C) The doses- and time-dependent effects of MMC on ER stress-associated proteins. Cells were treated with different concentrations of MMC, respectively, for 5 minutes and then cultured for 24 hours (phospho-IRE1 and IRE1 were detected at 9 hours) or treated with 0.4 mg/ml MMC for 5 minutes and cultured for different time intervals. The expression of GRP-78, P-IRE1, IRE1, P-PERK, PERK, P-eIF2α, eIF2α, CHOP, ATF6 and β-actin (loading control) was analyzed by Western blotting. (D) MMC-induced alternative splicing of XBP1 mRNA was examined via RT-PCR. The gel data shown in panels B, C and D are from experiments performed in triplicate with similar results (P- represents phospho-).

To gain more insight into the molecular effects of MMC-induced apoptosis, we measured the expression of GRP78 and CHOP. As detected via quantitative real-time PCR, GRP78 mRNA levels increased up to 6 hours after treatment and then declined slightly, while CHOP mRNA levels increased dramatically, and high levels were maintained up to 24 hours after treatment (Fig. 2A). Both proteins were upregulated in response to MMC treatment. However, GRP78 peaked at 6 hours, then decreased up to 24 hours (Fig. 2C).

We next investigated the effects of MMC on three ER stress transducers. All three pathways were activated in a dose-dependent manner within 24 hours of treatment (phospho-IRE1 and IRE1 were detected at 9 hours) (Fig. 2B). Regarding time-dependent kinetics (Fig. 2C), we observed a marked increase in phospho-IRE1 at 6 hours after treatment, which was sustained for the next 3 hours, and then declined to the initial level. Meanwhile, spliced XBP-1 mRNA, which represents a sequence-specific substrate that is cleaved by phospho-IRE1 [24], was monitored using RT-PCR (Fig. 2D). Consistent with the phospho-IRE1 data, the spliced form of the XBP-1 mRNA was generated over a similar time course after MMC treatment. These data indicate that the IRE1 pathway is temporarily induced by a single dose of MMC. ATF6 p90 decreased steadily from 6 to 24 hours after MMC treatment (Fig. 2C). However, the PERK pathway exhibited a distinctive feature. Western blot analysis showed that the levels of PERK, eIF2α, and CHOP increased with increasing time after treatment (Fig. 2C). Taken together, these results led us to conclude that ER stress is induced by MMC in human fibroblasts.

### Attenuation of MMC-induced Apoptosis by Suppression of CHOP/GADD153

**Figure 3:**
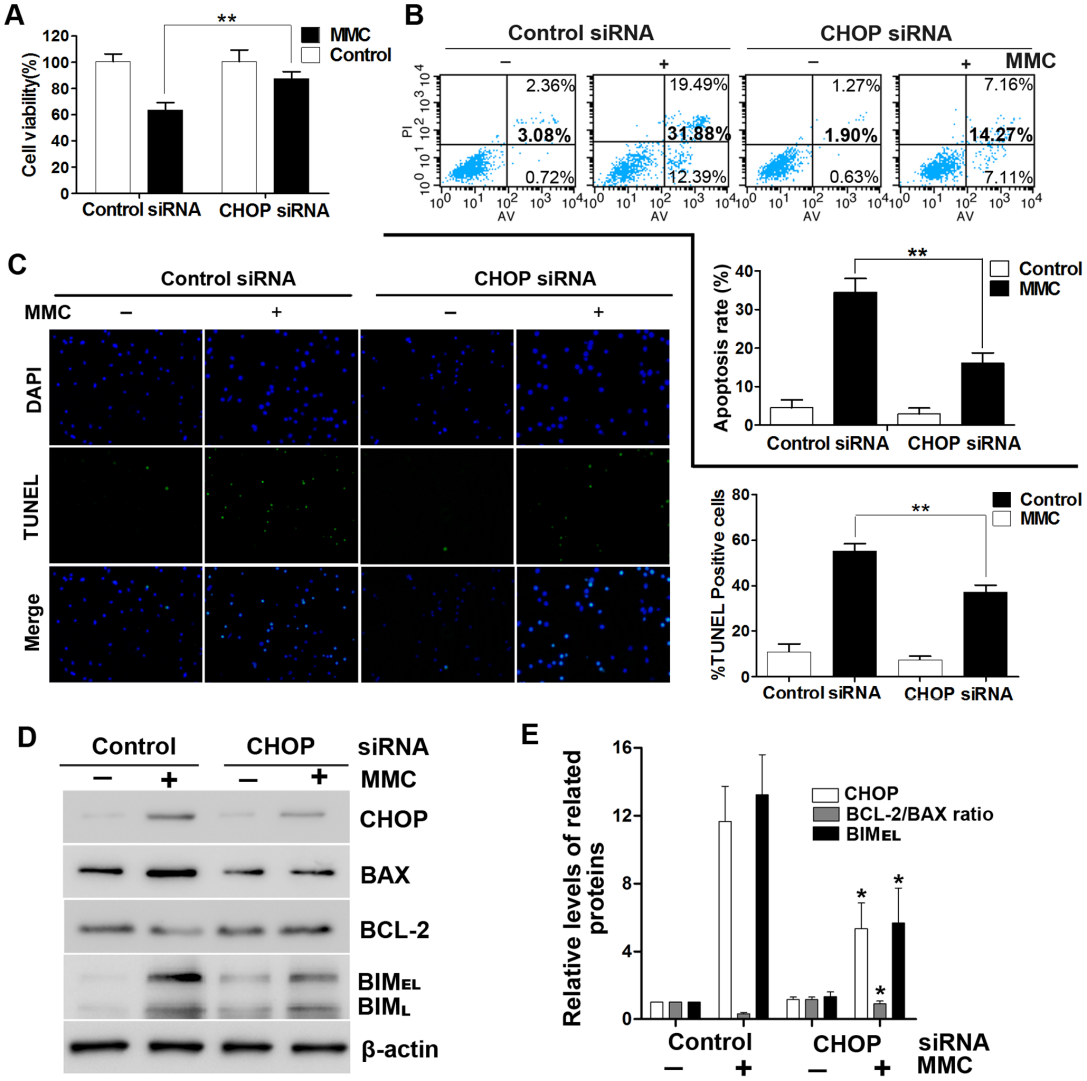
CHOP is essential for MMC-induced apoptosis. Fibroblasts were transfected with CHOP or control siRNA (non-targeting siRNA). After treatment with 0.4 mg/ml MMC for 48 hours, the cells were analyzed in a number of assays. (A) Cell viability was assessed with the CCK-8 assay. (B) Annexin V/propidium iodide double staining was performed to detect the apoptosis rate. (C) TUNEL-stained cells were observed under a fluorescence microscope. (D) Whole-cell lysates were used for Western blotting with antibodies specific for CHOP, BAX, BCL-2, BIM and β-actin (loading control). This experiment was performed in triplicate. (E) The band intensities for CHOP, BCL-2/BAX and BIM were expressed as a histogram relative to β-actin. The control group (no MMC treatment) was normalized to a value of 1.0-fold. The data in panels A, B, C and E are the mean ± SD of at least three independent experiments. *P<0.05, **P<0.01 versus the control group (with MMC treatment).

CHOP is well known for its proapoptotic role during ER stress [20], [25]. Therefore, we used siRNA to downregulate CHOP expression and confirm that CHOP is required for MMC-induced apoptosis in fibroblasts. After treatment with MMC (0.4 mg/ml) and incubation for 48 hours, the CCK-8 assay showed that CHOP knockdown effectively increased cell viability (38%) compared with the control group (Fig. 3A). Annexin V/propidium iodide double staining demonstrated that MMC induced 31.88% apoptosis in the control group, whereas only 14.27% of the cells in the CHOP knockdown group were apoptotic (Fig. 3B). TUNEL staining indicated that the percentage of TUNEL-positive cells decreased from 55.13% in the control group to 37.06% in the CHOP knockdown group (Fig. 3C). The relevant proteins were detected by Western blotting. Previous work has determined that BCL-2 and BIM are downstream of CHOP [26], [27]. Here, CHOP knockdown was observed to downregulate BIM expression 48 hours after MMC treatment. CHOP knockdown also increased BCL-2 and decreased BAX levels. The BCL-2/BAX ratio was upregulated 2.8-fold compared with the control siRNA group treated with MMC (Fig. 3D and 3E). Taken together, these data suggest that CHOP plays a crucial role in MMC-induced fibroblast apoptosis.

### The PERK Pathway Plays a Key Role in MMC-induced Apoptosis

The UPR transmits survival and apoptotic signals simultaneously and then executes cytoprotective or proapoptotic functions. Mild ER stress induces adaptation and survival, while strong or chronic ER stress leads to apoptosis [28], [29]. Previous studies have suggested that different pathways could be selectively activated in different environments. Persistent PERK signaling impairs cell proliferation and promotes apoptosis in many cell lines [30], [31].

**Figure 4:**
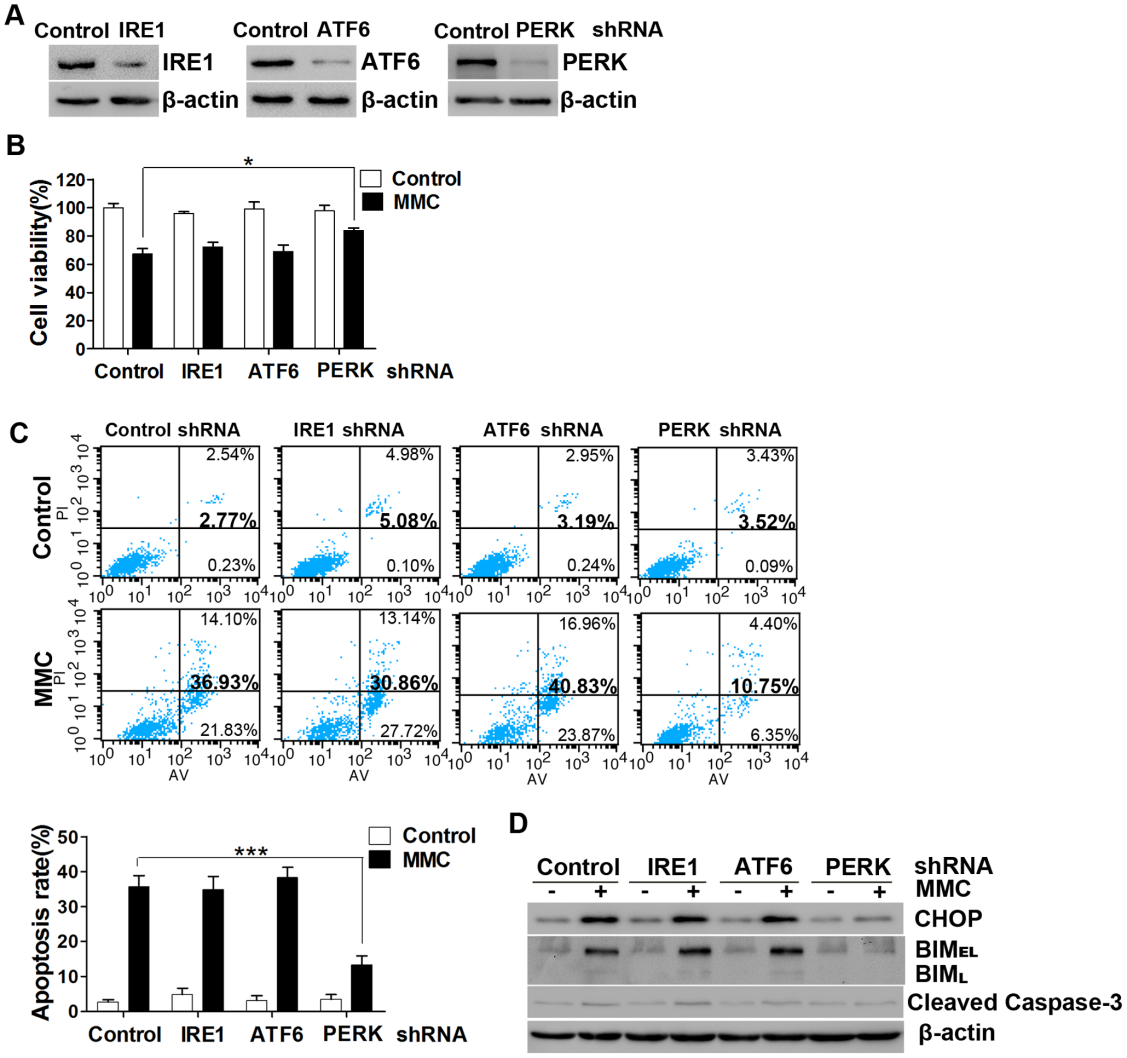
The role of three UPR sensors in MMC-induced apoptosis in fibroblasts. Fibroblasts were transfected with the PERK, ATF6, IRE1 or non-targeting lentiviral-mediated shRNAs. (A) The expression levels of the targeted transcripts were determined by Western blotting with PERK, ATF6, IRE1 and β-actin antibodies. The presented data represents the results of two independent experiments. (B) Transfected cells were treated with MMC as described in the text and then cell viability was measured after 48 hours. (C) Apoptosis rates were determined via Annexin V/propidium iodide double staining and are shown in the bar graph. (D) After MMC treatment (0.4 mg/ml, 5 minutes) and incubation for 48 hours, equal amounts of the whole cell lysates were analyzed by Western blotting with antibodies specific for CHOP, BIM, cleaved caspase-3, and β-actin (loading control). This experiment was performed in triplicate. The data presented in panels B and C are the mean ± SD of three independent experiments, *P<0.05, ***P<0.001.

To further investigate which pathways play pivotal roles in MMC-induced ER stress associated with apoptosis, we used lentiviral-mediated shRNAs to downregulate the expression of the three major ER stress sensors: PERK, IRE1, and ATF6. Lentiviruses that downregulated the three transducers strongly decreased the expression of the corresponding proteins in fibroblasts (Fig. 4A). These cells were incubated with MMC as described above. Cell viability was analyzed using the CCK-8 assay 48 hours after treatment. Silencing of PERK resulted in a subtle increase in cell viability and a decrease in cell apoptosis, whereas the IRE1 and ATF6 treatments resulted in no difference compared with cells transfected with control shRNA (Fig. 4B and 4C). The levels of CHOP, BIM, and cleaved caspase-3 were detected by Western blotting 48 hours after MMC treatment. As expected, decreased CHOP, BIM, and cleaved caspase-3 were only detected in the PERK knockdown group (Fig. 4D). These observations suggest that CHOP is downstream of PERK, rather than the other two pathways examined here.

### The IRE1 Pathway is not Essential for MMC-induced JNK Activation or the JNK Apoptosis Pathway

**Figure 5:**
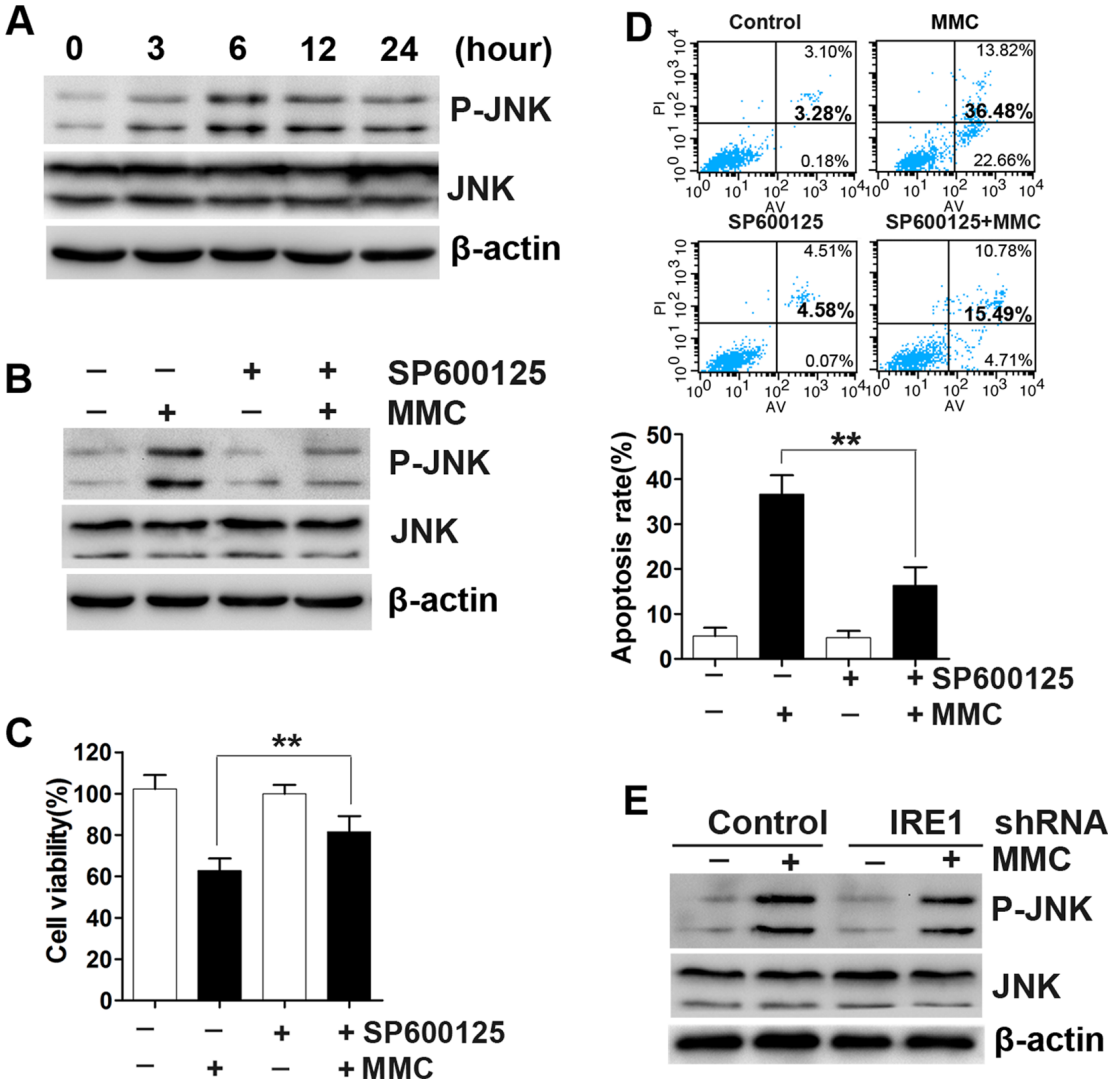
The IRE1 pathway is not necessary for MMC-induced JNK activation. (A) JNK activation in response to MMC treatment (0.4 mg/ml, 5 minutes) was detected at various times by Western blotting. (B) Fibroblasts were pretreated with a 5 µM concentration of the JNK inhibitor SP600125, then treated with MMC (0.4 mg/ml, 5 minutes). The expression of P-JNK, JNK, and β-actin (loading control) was measured by Western blotting 24 hours after MMC treatment. (C) Cell viability and (D) apoptosis rates were quantified using CCK-8 kit assays and Annexin V/propidium iodide double staining, 48 hours after MMC treatment. The histograms represent the mean ± SD of three independent experiments. **P<0.01. (E) Fibroblasts were transfected with encoded IRE1 shRNA or non-targeting shRNA (control shRNA) and then treated with MMC (0.4 mg/ml, 5 minutes). Whole cell lysates were prepared 24 h after MMC treatment and subjected to Western blotting to detect P-JNK, JNK and β-actin (loading control). The results from the gels shown in panels A, B and E are from experiments performed in triplicate with similar results.

The IRE1/apoptosis signal-regulating kinase 1(ASK1)/JNK pathway has been linked to apoptosis activation following irreversible ER stress [32], [33]. Therefore, we examined the relationship between the JNK and IRE1 pathways. First, we demonstrated that MMC could induce JNK activation (Fig. 5A). The JNK inhibitor SP600125 reduced the MMC-induced increase in phospho-JNK (Fig. 5B) and antagonized MMC cytotoxicity by restoring cell viability and decreasing the apoptosis rate compared with the SP600125-only group (Fig. 5C and 5D). These results indicate that MMC-induced JNK activation is another apoptosis pathway in fibroblasts. It is widely known that JNK functions downstream of IRE1, so we next determined whether the IRE1/ASK1/JNK pathway is required for MMC-induced fibroblast apoptosis. We measured the activities of phospho-JNK under IRE1 knockdown and in control cells. We found that downregulation of IRE1 did not affect MMC-induced phospho-JNK expression (Fig. 5E). These results indicate that the IRE1 pathway may not participate in the activation of JNK. Therefore, there may be other pathways that are responsible for JNK activation.

### MMC Induces ROS Production and Links Oxidative Stress and ER Stress

**Figure 6:**
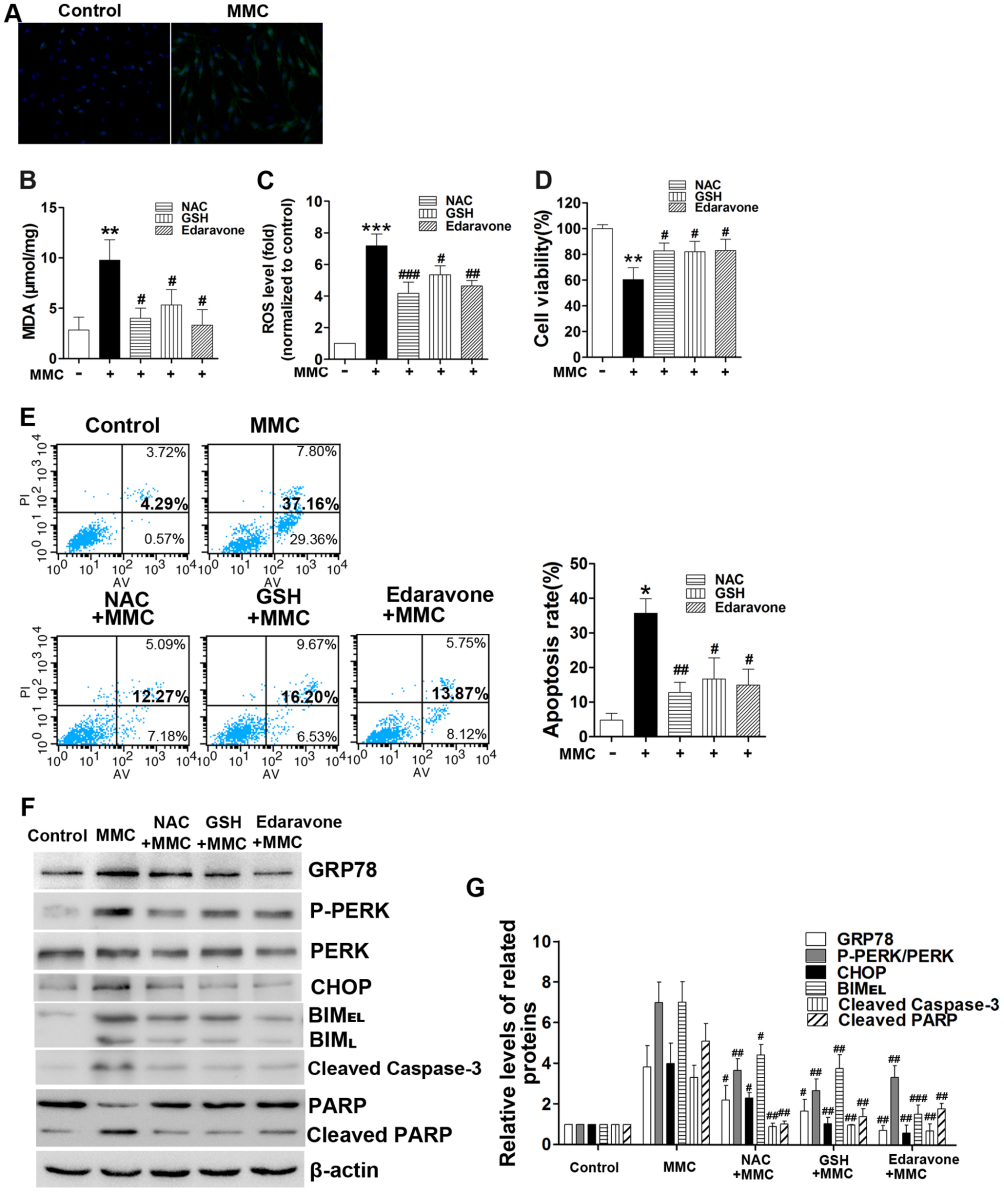
Increases in ROS induced by MMC triggers ER stress that can be blocked by antioxidants. (A) ROS levels were detected by using DCFH-DA and observed via fluorescence microscopy (200x magnification) 48 hours after MMC treatment. Nuclei are shown in blue and ROS staining in green. The fibroblasts were pretreated with 10 mM NAC, 10 mM GSH and 10 µM edaravone for 2 hours, respectively, then treated with MMC (0.4 mg/ml, 5 minutes) and incubated for 48 hours. (B) MDA generation was measured and is shown in the histogram. (C) The mean of DCF fluorescence intensity, which is indicative of ROS generation, was measured via flow cytometry and is depicted graphically as the relative fold increase of the control. (D) Cell viability and (E) apoptosis rates were examined 48 hours after MMC treatment using CCK-8 assays or Annexin V/propidium iodide double staining. (F) Western blot analysis of related proteins in fibroblasts 24 hours after MMC treatment. The expression of GRP-78, P-PERK, PERK, CHOP, BIM, cleaved caspase-3, PARP, cleaved PARP and β-actin (loading control) was analyzed. (G) The band intensities for GRP-78, P-PERK, PERK, CHOP, BIM, cleaved caspase-3, and cleaved PARP are shown as a histogram. The control group without MMC treatment was normalized to a value of 1.0-fold. The gel data results from the gels come from experiments performed in triplicate with similar results. The presented bar graphs shown in panels B, C, D and E are the average results from three different experiments. *P<0.05, **P<0.01, ***P<0.001 versus the control group (without MMC), ^#^P<0.05, ^##^P<0.01, ^###^P<0.001 versus the MMC treatment group (without antioxidants).

Previous studies have reported antitumor effects of MMC through ROS generation [13], [14], [15], [16]. However, our method of MMC administration is different from that in tumor cells. To determine whether MMC could cause oxidative stress in fibroblasts, ROS and MDA levels were measured after MMC treatment. Three anti-oxidants: N-acetylcysteine (NAC), glutathione (GSH), and edaravone, were used to treat fibroblasts. All of these compounds have been widely accepted as efficient oxygen radical scavengers [34], [35], [36]. Cells pretreated with anti-oxidants and then subjected to MMC treatment for 48 hours were analyzed to determine their ROS levels via fluorescence microscopy and flow cytometry. We observed an increase in the percentage of ROS-positive cells (stained with green fluorescence) in the MMC-treated group (Fig. 6A). We also measured MDA, which is generated by ROS-induced lipid oxidation [37]. The MDA level also increased 48 hours after MMC treatment (Fig. 6B). In the groups exposed to anti-oxidants, we observed a dramatic decrease in ROS and MDA levels (Fig. 6B and Fig. 6C). These results indicate that treatment with MMC elevates ROS levels.

To further confirm the role of oxidative stress in MMC-induced apoptosis, cell viability and the rate of apoptosis were monitored 48 hours after MMC treatment in the presence or absence of anti-oxidants. MMC treatment induced an inhibition of proliferation and promotion of apoptosis, whereas these effects were inhibited by NAC, GSH, and edaravone (Fig. 6D and Fig. 6E). These results indicate that ROS generation plays an important role in MMC-induced fibroblast apoptosis. There is accumulating evidence that suggests a link between ROS production and ER stress [22], [38]. Furthermore, the PERK pathway is involved in ROS-mediated ER stress [39]. We therefore assessed the MMC-induced ER stress-related proteins GRP78, CHOP, and PERK under normal and anti-oxidant-pretreated conditions. BIM, cleaved caspase-3 and cleaved-PARP were also detected as signals of apoptosis. We found that the expression of these proteins increased 24 hours after MMC treatment. However, NAC, GSH, and edaravone pretreatment significantly attenuated the MMC-induced upregulation of phospho-PERK, GRP78, and CHOP. BIM expression was also inhibited by these anti-oxidants. Additionally, cleaved caspase-3 and PARP decreased to their initial levels (Fig. 6F and 6G). These results suggest that ROS-mediated ER stress is involved in the MMC-induced apoptosis of human fibroblasts.

## Discussion

This study reveals several important findings regarding MMC-induced apoptosis in fibroblasts for the first time to our knowledge. First, treatment with MMC for 5 minutes led to a significant increase in fibroblast apoptosis in a dose- and time-dependent manner. Second, MMC-induced fibroblast apoptosis was accompanied by the activation of ER stress-responsive elements, and the increase in apoptosis was attenuated by CHOP deletion. Third, PERK is required for MMC-induced fibroblast death mediated by CHOP. Exposure to MMC for 5 minutes using a single application of 0.4 mg/ml results in significant toxicity to fibroblasts, inhibiting cell viability (as detected in CCK-8 assays) and increasing the numbers of apoptotic and necrotic cells (as detected by TUNEL staining and Annexin V/propidium iodide double staining). Furthermore, MMC significantly increased ROS generation, which can lead to DNA cleavage and the generation of a variety of DNA lesions [40]. DNA damage is often accompanied by cell cycle arrest and apoptosis [41]. In this study, cell cycle analysis via flow cytometry showed that MMC could cause cell cycle arrest in G1 phase. We also found that MMC increased PARP and caspase-3 cleavage, both of which are involved in DNA damage [42]. These data suggest that the MMC-induced G1 phase arrest and apoptosis observed in fibroblasts is a consequence ROS generation and DNA damage, consistent with previously published data from tumor cells and fibroblasts [16], [43], [44], [45], [46].

The ER responds to stress conditions by activating a range of stress response signaling pathways, which are referred to as the UPR. Three proximal sensors (IRE1, PERK, and ATF6) act in concert to regulate the UPR through their respective signaling cascades [47]. The protein chaperone GRP78/BiP is the master regulator of these pathways. With the accumulation of unfolded proteins, GRP78/BiP is released from IRE1 and permitted to dimerize, which activates its kinase and RNase activities to initiate XBP1 mRNA splicing, thereby producing a potent transcriptional activator. Similarly, GRP78/BiP release from ATF6 (ATF6 p90) permits ATF6 transport to the Golgi compartment, where it is cleaved to yield a cytosolic fragment (ATF6 p50) that migrates to the nucleus to further activate the transcription of UPR-responsive genes. Finally, GRP78/BiP release enables PERK dimerization and activation to phosphorylate eIF2α, and eIF2α phosphorylation induces the translation of ATF4 mRNA [47]. In this study, MMC was found to upregulate GRP78/BiP and its downstream transcription factor CHOP. IRE1 activation and XBP-1 mRNA splicing also increased, while ATF6 p90 levels decreased. Furthermore, PERK phosphorylation and PERK-mediated phosphorylation of eIF2α also increased. These data suggest that MMC activates the UPR and causes cell death through the induction of apoptosis in fibroblasts.

An increase in the expression of the transcription factor CHOP results in the downregulation of BCL-2 and upregulation of BAX [48], [49]. In the present work, we showed that fibroblasts in which CHOP is knocked down via siRNA treatment are more resistant to MMC-induced apoptosis than wild-type cells. These cells also exhibit upregulation of BCL-2 and downregulation of BAX and BIM, suggesting that CHOP is induced and plays a role in MMC-mediated fibroblast apoptosis.

CHOP is a non-ER-localized transcription factor that is induced by a variety of adverse physiological and pharmacological conditions, including ER stress. ER stress regulates CHOP transcription through two UPR pathways (PERK and ATF6 or XBP-1). The CHOP promoter contains an ERSE, which responds to agents that activate the mammalian UPR pathway, and a C/EBP-ATF composite site that has been shown to regulate CHOP transcription in response to a variety of other stress conditions. Activation of PERK leads to the phosphorylation of eIF2α and synthesis of ATF4. ATF4 together with C/EBP-ß, bind to the composite site and transactivate the CHOP promoter [50], [51].

In the present work, we found that PERK activation and eIF2α phosphorylation were important for the MMC-mediated upregulation of CHOP in fibroblasts. PERK depletion was observed to decrease CHOP expression and caspase-3 cleavage in MMC-treated fibroblasts, demonstrating the importance of this pathway. The second pathway is activated when ATF6 is cleaved by S1P and S2P, and cleaved ATF6 translocates to the nucleus. Together with NF-Y, processed ATF6 transactivates the CHOP and ER chaperone promoters by binding to their respective ERSEs [51]. However, Yoshida and colleagues were unable to detect binding of endogenous ATF6 or XBP-1 to this site after stress [52]. In the present study, the depletion of ATF6 and IRE1 had no effect on CHOP expression in MMC-treated fibroblasts, indicating that the effect of MMC on CHOP upregulation is not due to the activation of ATF6 and IRE1.

A second cell death signaling pathway that is activated by ER stress is mediated by IRE1. Activated IRE1 recruits tumor necrosis factor receptor–associated factor 2 (TRAF2) to elicit JNK phosphorylation and activation. The IRE1 cytoplasmic domain interacts with the adaptor protein TRAF2. IRE1 and TRAF2, in turn, interact with MAPK (p38MAPK/JNK) and ASK1, which subsequently phosphorylate and activate JNK [32], [33], [53]. TRAF2 couples the activation of death receptors at the plasma membrane to the activation of Jun kinase (JNK) and stress-activated protein kinase (SAPK), in addition to associating with caspase-12 and regulating its activation [33], [53]. It is well known that human caspase-12 has acquired mutations that are not expressed in most human cells [54]. In the present study, neither TRAF2 nor caspase-12 was detected in MMC-treated fibroblasts (data not shown). In addition, the activation of JNK by MMC treatment was not impaired in IRE1-depleted cells. These findings indicate that MMC-induced apoptosis in fibroblasts does not involve activation of IRE1.

Previous studies have suggested that there is an association between protein folding and ROS generationthat ultimately results in protein misfolding in the ER [22], [38]. In addition, several reports have revealed that ER stress occurs in response to oxidative stress during some apoptotic processes triggered by albumin and cadmium in proximal tubular epithelial cells [55], [56]. ROS have also been implicated in MMC-induced apoptosis in cell culture systems and animal models. In this study, the exposure of fibroblasts to MMC caused a notable increase in ROS production and apoptosis following a single dose of MMC. Moreover, treatment with antioxidants (NAC, GSH and edaravone) prevented MMC-induced ER stress and CHOP in fibroblasts in a process mediated by their radical-scavenging activity. Therefore, MMC may induce apoptosis in fibroblasts via oxidative stress-regulated, endoplasmic reticulum stress-triggered signaling pathways.

In conclusion, this study suggests that the endoplasmic reticulum stress response is involved in MMC-induced apoptosis in fibroblasts, which may be mediated by ROS. Thus, this study offers new insight into the prevention of postoperative scarring after MMC treatment.
